# Heavy Metal Bioaccumulation, Growth Characteristics, and Yield of *Pisum sativum* L. Grown in Agricultural Soil-Sewage Sludge Mixtures

**DOI:** 10.3390/plants9101300

**Published:** 2020-10-01

**Authors:** Ebrahem M. Eid, Ahmed F. El-Bebany, Mostafa A. Taher, Sulaiman A. Alrumman, Tarek M. Galal, Kamal H. Shaltout, Nasser A. Sewelam, Mohamed T. Ahmed

**Affiliations:** 1 Biology Department, College of Science, King Khalid University, Abha 61321, Saudi Arabia; ebrahem.eid@sci.kfs.edu.eg (E.M.E.); mostafataherahm@yahoo.com (M.A.T.); salrumman@kku.edu.sa (S.A.A.); mtahmed@kku.edu.sa (M.T.A.); 2 Botany Department, Faculty of Science, Kafrelsheikh University, Kafr El-Sheikh 33516, Egypt; 3 Plant Pathology Department, Faculty of Agriculture, Alexandria University, El-Shatby, Alexandria 21545, Egypt; 4 Botany Department, Faculty of Science, Aswan University, Aswan 81528, Egypt; 5 Botany and Microbiology Department, Faculty of Science, Helwan University, Cairo 11795, Egypt; tarek_abosarea@science.helwan.edu.eg; 6 Biology Deprtment, College of Science, Taif University, Taif 21974, Saudi Arabia; 7 Botany Department, Faculty of Science, Tanta University, Tanta 31527, Egypt; kamal.shaltout@science.tanta.edu.eg (K.H.S.); sewelam@science.tanta.edu.eg (N.A.S.)

**Keywords:** pea, soil amendment, accumulation, metal translocation, fertilization

## Abstract

The application of sewage sludge (SS) in agriculture is an alternative disposal method for wastewater recycling and soil fertilization. This study evaluated heavy metal bioaccumulation, growth, and yield of *Pisum sativum* (pea) grown in agricultural soil amended with SS at rates of 0, 10, 20, 30, and 40 g/kg. The results show that root, shoot, pod length, biomass, and number of leaves and pods increased with SS amendments of 10 and 20 g/kg, while rates declined at 30 and 40 g/kg. SS had greater salinity and organic content than the soil. Heavy metals in the postharvest soil samples increased for all SS application rates except Fe and Mo. The significant increase in Cd content started at the lowest amendment rate 10 g/kg; for Co, Mn, and Pb, the significant increase was detected at the highest amendment rate (40 g/kg). Generally, all heavy metals increased significantly in portions of *P. sativum* except Cd in the shoot. At an amendment rate of 10 g/kg, Co in the shoot and root, Cr in the fruit, Cu in the root, Fe in the fruit, Mn in the shoot and fruit, Mo in the fruit, Pb in the shoot, and Zn in the fruit were elevated significantly. In contrast, the concentrations of Cd in the fruit, Cr in the root, Cu in the shoot, Fe in the shoot and root, Ni in the fruit and root, Pb in the fruit and root, and Zn in the root significantly increased only at the highest rate of 40 g/kg. The highest regression *R*^2^ was 0.927 for Mn in pods and the lowest was 0.154 for Cd in shoots. Bioaccumulation and translocation factors were > 1 for Mo and the bioaccumulation of Pb was >1. SS could be used for pea fertilization but only at rates below 20 g/kg to avoid environmental and health hazards.

## 1. Introduction

The rapid expansion of population in several countries increases pressure on the agriculture to produce more food. Low soil fertility in Saudi Arabia and several surrounding countries plays a role in the decline of crop production. On the edges of towns and cities, there are usually some rural areas that produce daily vegetables. Sewage sludge (SS) provides agricultural soils with organic matter and several macro- and micronutrients which are used as fertilization materials [1], and its amendment, inducing alteration of soil pH, affects the bioavailability of several elements. For these reasons, the application of SS is considered a low-cost waste disposal method reducing crop production costs [2].

Numerous investigations have studied the influence of municipal SS application on many crops in terms of their growth, productivity, and metal uptake. Investigated crops include trees such as olive [3], field crops such as alfalfa [4], rice [5], corn [6], wheat [7,8,9,10], barley [11,12], cotton [13], and broad bean [14], vegetable crops such as French bean [15], cucumber [16,17], cherry tomato [18], sweet pepper [19], cabbage and broccoli [20], as well as spinach [21,22,23]. However, variations in SS source and composition, agricultural soil type, and environmental conditions require more specific estimates for different cropping systems [24]. Heavy metal content in SS is a limiting factor for SS application due to the toxic effects of these metals on plants. Heavy metals phytotoxicity includes alteration of pigment biosynthesis, photosynthesis process, inactivation of enzymes, nutrients assimilation and plant hormonal balance [25,26,27]. Monitoring heavy metals in plants growing in soils amended with SS is an important issue as the accumulation of heavy metals may contaminate the food chain through entering animal and human bodies, causing severe health disorders [25].

Pea (*Pisum sativum* L.) is a legume plant and member of the family Fabaceae. It is an important pulse crop that provides human and animals with nutritional values. *P. sativum* is considered a cheap source of protein (average of 20–22% protein per seed) in developing countries [28]. Several studies demonstrated that sewage irrigation improved the nodulation on the root system of *P. sativum* but suppressed the colonization of mycorrhiza [29]. Germination of *P. sativum* and other legume seeds was negatively affected by industrial wastewater applications [30]. Previous experiments, considering SS’s impact on another legume, *Faba sativa*, indicated a dose-dependent improvement in growth and yield starting with increasing the biomass and yield followed by decline at high concentrations of SS amendments [14]. Interestingly, SS compost has been reported as suppressing the damping-off disease (caused by *Rhizoctonia solani* and *Pythium ultimum*) in *P. sativum* [31]; however, the effect of SS compost differs according to the crop and the associated pathogen type [32].

The objectives of this research were to assess the impact of SS amendments to agricultural soil on the growth and biomass of pea, and to determine the bioaccumulation and transformation of heavy metals in different portions of pea plants.

## 2. Results and Discussion

### 2.1. Chemical Analyses of Sewage Sludge and Agricultural Soil

The data in Table 1 show the chemical analysis of both SS and cultivated fields’ soil before mixing. SS had higher salinity than the agricultural soil, and had an organic matter content approximately 65 times more than that of the soil. The agricultural soil was more alkaline than the SS (which was neutral, pH 6.98). High organic matter content in the SS resembled previous analyses on other plants from the same municipal station [14]. The permissible and average normal limits of heavy metals in SS and agricultural soil are shown in Table 1. Heavy metal analysis showed that SS had higher values of Cr, Cu, Ni, Pb, and Zn than the agricultural soil. However, Fe, Mn, Cd, and Co content was higher in the cultivated soil. Although all the measured heavy metals in SS were lower than the permissible limits [33], the additive effect of using SS in agricultural soil should be monitored in order to keep the content of these metals within the average normal limits [34]. 

### 2.2. Plant Growth Measurements

**Figure 1:**
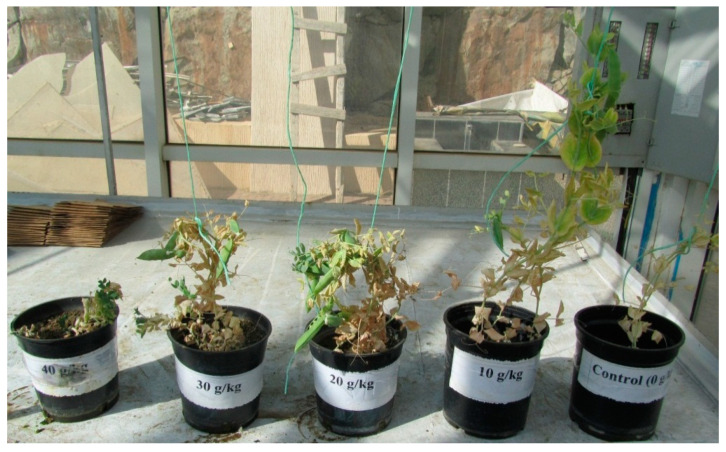
A demonstration of five pots (replicates) represents the *Pisum sativum* plants grown in sewage sludge-soil with amendment rates of 0 (control), 10, 20, 30 and 40 g/kg.

**Figure 2:**
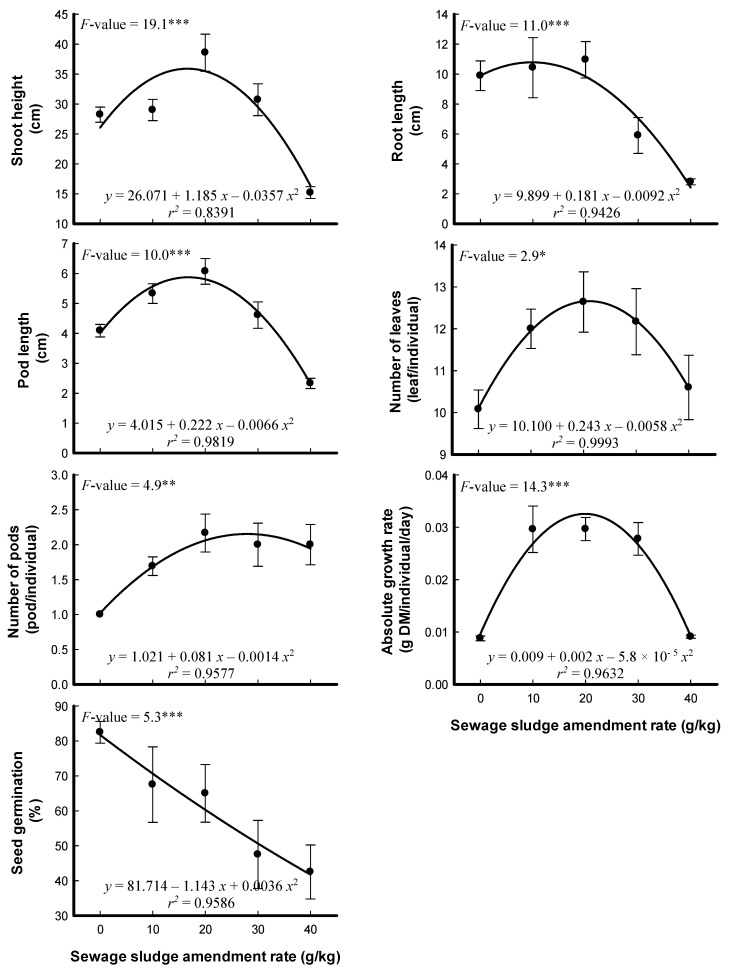
Effects of different sewage sludge amendment rates on the morphometric parameters of *Pisum sativum* that had been grown for 57 days (means ± standard error, *n* = 6). The *F*-values represent one-way analysis of variance (ANOVA) and a degree of freedom (*df*) = 4. *: *p* < 0.05, **: *p* < 0.01, ***: *p* < 0.001.

**Figure 3:**
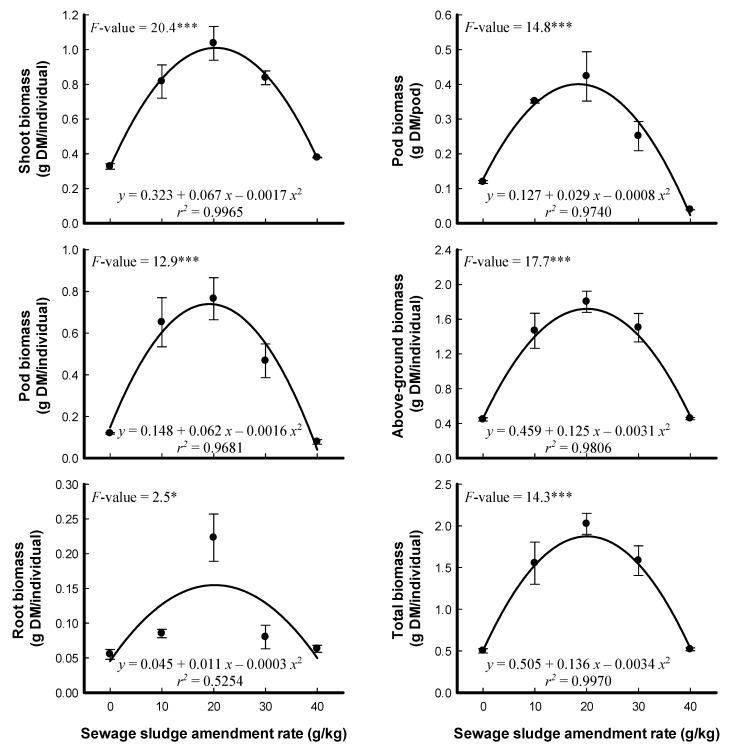
Effects of different sewage sludge amendment rates on the biomass of *Pisum sativum* that had been grown for 57 days (means ± standard error, *n* = 6). *F*-values represent a one-way ANOVA and degrees of freedom (*df*) = 4. *: *p* < 0.05, ***: *p* < 0.001.

The effects of different SS amendment rates on the morphology and morphometric measurements of *P. sativum* are illustrated in Figure 1 and Figure 2. Germination of *P. sativum* seeds was negatively affected at all SS amendment rates. The effects on the germination percentage appear to be related to heavy metal concentration in SS. Cr and Cu have been reported as inhibitors of *P. sativum* germination, and they also suppressed the germination and seedling growth of wheat [25,35,36]. However, root, shoot, and pod length, number of leaves and pods, and absolute growth rate of the plant gradually increased at low rates of SS amendments, followed by decline at amendment rates of 30 and 40 g/kg (Figure 2). The same trend was observed for root, shoot, pod, above-ground and total biomasses of *P. sativum* (Figure 3). The enhancement of *P. sativum* growth and yield may be due to the organic materials and micronutrients in the low levels of SS amendments [28]. On the contrary, using SS compost did not increase the pea yield but did increase the yield of the monocot plants triticale and maize [1]. The reduction in pea growth at high rates of SS amendments could be due to nutrient deficiency. The antagonistic or synergistic effect of heavy metals on uptake of elements was reported in many studies; Cd inhibited the absorption and accumulation of K, Ca and Fe [37,38]. It has been reported that similarities of heavy metals with nutrients cations result in competition for absorption by root cells [25,26]. At the high rates of SS amendments, heavy metals enter into the cells of plant roots and may affect the primary metabolic pathways through direct interaction with sulfhydryl groups of proteins, which consequently reduces plant growth [39]. The generation of reactive oxygen species in plant cells as a response to heavy metals may cause damage to macromolecules such as proteins and lipids, which results in the reduction in plant growth [27]. In addition, the decline in plant biomass may refer to the SS salinity and/or inhibition of growth hormones biosynthesis in plants grown in soil with a high content of SS [40].

### 2.3. Postharvest Chemical and Heavy Metal Analyses of Soil and Pea Portions

The chemical analysis of samples collected from soil for all SS treatments after *P. sativum* harvesting is shown in Table 2. A significant increase in salinity was detected at amendment rates of 30 and 40 g/kg and the organic matter was increased significantly under all amendment rates, whereas pH significantly decreased until reaching 7.09 at an amendment rate 40 g/kg. Heavy metals in the postharvest soil samples increased for all SS application rates except Fe and Mo. The significant increase in Cd content started at the lowest amendment rate 10 g/kg; for Co, Mn, and Pb, the significant increase was detected at the highest amendment rate (40 g/kg). The measured heavy metals in the soil mixtures in all treatments after harvesting pea plants were within the allowed limits reviewed in several studies [34,41,42]. Similar effects of SS amendments on cultivated soil chemical properties were observed in our previous studies on wheat, faba bean, and spinach [10,14,23].

The concentrations of heavy metals in roots, shoots, and fruits of *P. sativum* grown for 57 days in the soils mixed with SS are shown in Table 3. Generally, all heavy metals increased significantly in portions of *P. sativum* except Cd in the shoot. At an amendment rate of 10 g/kg, Co in the shoot and root, Cr in the fruit, Cu in the root, Fe in the fruit, Mn in the shoot and fruit, Mo in the fruit, Pb in the shoot, and Zn in the fruit were elevated significantly. In contrast, the concentrations of Cd in the fruit, Cr in the root, Cu in the shoot, Fe in the shoot and root, Ni in the fruit and root, Pb in the fruit and root, and Zn in the root significantly increased only at the highest amendment rate. Heavy metals were reported to be accumulated in all parts of plants such as peanut [43] and pea [44]. The results indicate that the heavy metal accumulation in pea portions was below the safe limit set by Codex Alimentarious Commission [45], except for Cr, Fe, Mo, Ni, Pb and Zn in roots. The linear regression estimations of heavy metal content in the different portions of *P. sativum* over all SS amendment rates indicated that the highest *R*^2^ values were 0.927 and 0.904 for Mn and Cr in fruits, respectively. The lowest *R*^2^ values were 0.157 and 0.154 for Mn in roots and Cd in shoots, respectively (Table 4). Translocation of heavy metals by *P. sativum* has been detected in the case of using electroplating industrial sludge that was acidic (like the municipal SS used in this study). The addition of 0.5% lime as a treatment to the SS minimized the uptake and translocation of the toxic metals in *P. sativum* plants [46].

### 2.4. Bioaccumulation and Translocation of Heavy Metals in Pea

BF and TF of heavy metals in harvested *P. sativum* were calculated (Table 5). TF were assessed for both shoot and fruits, since the plant shoot system could be used as feeding materials for livestock and the fruits are used for human food. The accumulation of Cd and Cr was significant only at high SS amendment rates (30 and 40 g/kg). Co, Ni, and Pb did not accumulate significantly at any treatment level. The beneficial micronutrient metals such as Fe and Zn accumulated at a treatment of 40 g/kg. Mn accumulation was not significant. The TF of heavy metals in the shoot system increased at high amendment rates for Co, Cr, Mn, Mo, Ni, and Zn. For the fruits, the TF of Ni, Pb, and Zn were not significant at any treatment level, however, Co, Cr, Fe, Mn, and Mo translocate into the fruits at the 30 g/kg amendment rate. Bioaccumulation and translocation factors were >1 for Mo and the bioaccumulation of Pb was >1 (Table 5). Accumulation of heavy metals in plant shoots at high SS amendment rates interferes with essential physiological processes such as photosynthesis and subsequently affects the growth and yield of the pea plant [36]. Accumulation of heavy metals in the aboveground portions of plants may affect the carbon dioxide fixation and nitrogen metabolism which, ultimately, decrease the growth and development of the plant [25]. Prediction models of heavy metal concentrations in *P. sativum* grown in soils amended with sewage sludge were published recently by our research group [47]. 

The results indicate that SS could be used to improve agricultural soil quality only with low amendment rates of 10 and 20 g/kg. Frequent use of SS should be taken in account for long-term environmental and health hazard management.

## 3. Materials and Methods 

### 3.1. Plant Materials, Sewage Sludge Treatments, and Experimental Design

Seeds of *Pisum sativum* (Mashhour Company, Menoufiya Governorate, Egypt) were obtained from a local market in Abha city, Saudi Arabia. The agricultural soil used in the experiment was collected at a depth of 0–20 cm from neighboring cultivated fields. The SS was obtained from the Abha municipal sewage treatment plant (AMSTP), Aseer region, Saudi Arabia. The AMSTP treats around 41,275 m^3^ of urban wastewater per day using aerobic tertiary treatment, and the equivalent dry sewage sludge production was assessed as 90 tons per day with daily dumping. The agricultural soil and SS samples were prepared as described previously [16]. The experiment was performed in the greenhouse of the Biology Department, King Khalid University, Abha, Saudi Arabia.

The SS was mixed with agricultural soil at rates of zero (the control soil), 10, 20, 30, and 40 g/kg. Each treatment consisted of 6 replicates of plastic pots (6 L volume) filled with 4 kg/pot of the respective treatment. Ten seeds of *P. sativum* were planted in each pot. The experimental units were arranged in a randomized design. The plants were grown for 57 days (starting from 4 January 2018) in the greenhouse with a natural day/night regime and irrigated as needed and weed controlled manually. After 15 days, the plants were counted and then manually thinned to one plant/pot.

### 3.2. Plant Morphology and Biomass Measurements

The shoot heights and root and pod lengths were measured, and the number of leaves and fruits (pods) per individual were counted. The germination percentage was calculated by dividing the germinated seed in each pot by the total seed used in planting. The plant materials were washed under running water, and divided into roots, pods, and shoots (stems and leaves). The partitioned plant materials were dried at 60 °C for one week and then ground in a plastic mill and stored. The above-ground biomass refers to the aerial portions of the plant, i.e., the sum of the pod and shoot biomass, while the total biomass refers to the sum of the shoot and root biomass. The absolute growth rate (AGR) was obtained as described by Radford [48].

### 3.3. Sample Analysis

For physicochemical analyses, the soil and SS samples were air-dried for two weeks and then ground and sieved through a 2 mm sieve. The dried samples were analyzed for organic matter content using a loss-on-ignition method at 550 °C for 2 h. [49]. Electrical conductivity (EC) and pH were measured in soil-water extracts at 1:5 [50]. For quantifying heavy metals (in 0.5–1.0 g of each sample), soil, SS or plant samples were digested using a mixed-acid digestion method (HNO_3_ and HClO_4_; 3:1, v/v). A microwave sample preparation system was used for digestion (PerkinElmer Titan MPS, PerkinElmer Inc., Waltham, MA, USA). Blank samples were included to confirm procedure accuracy. Various heavy metals, including Co, Cu, Fe, Mn, Ni, Zn, Cd, Cr, and Pb, were determined by inductively coupled plasma optical emission spectrometry (ICP-OES) (Thermo Scientific iCAP 7000 Plus Series; Thermo Fisher Scientific, Waltham, MA, USA) according to procedures outlined by [50]. The detection limits of heavy metals (in µg L−1) were as follows: 6.0 for Ni; 2.0 for Co, Cr, and Cu; 1.0 for Fe, Pb, and Zn; 0.3 for Mn; and 0.1 for Cd. The instrument setting and operational conditions followed the manufacturer’s specifications. Standard solutions with known concentrations of different heavy metals were prepared for the standardization of the system. A certified reference (SRM 1573a, tomato leaves) was used to verify the accuracy of the heavy metal determination. This reference material was digested and analyzed using the same methods as those applied to the *P. sativum* samples. Three independent biological replicates were performed for digestion and heavy metal quantification. Accuracy was estimated by comparing the measured concentration with the certified value and then expressed as a percentage. The recovery rates were 94.9% for Cd with a relative standard deviation (RSD) = 4.3%, 104.4% for Co (RSD = 8.0%), 95.7% for Cr (RSD = 6.2%), 101.6% for Cu (RSD = 3.3%), 102.9% for Fe (RSD = 4.9%), 97.1% for Mn (RSD = 7.4%), 98.9% for Mo (RSD = 3.8%), 95.2% for Ni (RSD = 9.0%), 103.5% for Pb (RSD = 5.6%) and 97.5% for Zn (RSD = 4.6%). The bioaccumulation factor (BF) and the translocation factor (TF) were calculated following the method of Ghosh and Singh [51]. BF is the concentration of a heavy metal (HM) in the roots (mg/kg)/concentration of the same HM in soil (mg/kg). TF is the concentration of an HM in the above-ground tissue (mg/kg)/concentration of the same HM in the roots (mg/kg).

### 3.4. Data Statistical Analyses

The data were examined for their homogeneity of variance and normality of distribution and, when required, the data were log-transformed before a one-way analysis of variance (ANOVA) was performed. Significant differences in the soil characteristics, biomass, plant morphometric parameters, and heavy metals (HMs) data for the *P. sativum* tissues, BFs, and TFs across all treatments, and the variation in BFs and TFs within 10 HMs through the same amendment rate of SS, were evaluated using one-way ANOVA. Tukey’s honest significant difference (HSD) test at *p* < 0.05 was used to identify the significant differences among the means of all treatments. A quadratic regression analysis [52] was performed to evaluate the response of the biomass and plant morphometric parameters of *P. sativum* grown in soils amended with different rates of SS. To assess the statistical relationships among the HM concentrations in *P. sativum* tissues (mg/kg) and the amendment rate of SS (g/kg), regression procedures were applied. Statistica 7.1 was used to perform all statistical analyses [53].

## 4. Conclusions

Sewage sludge (SS) application increases agricultural soil’s fertility and plant productivity when used in a suitable rate of soil amendment. The current study focused on the assessment of evaluating heavy metal bioaccumulation, growth, and yield of the legume crop *P. sativum* grown in agricultural soil mixed with SS at rates of zero, 10, 20, 30, and 40 g/kg. SS amendments affected seed germination of *P. sativum* severely. The inhibition of seed germination could be due to heavy metal content in SS. Heavy metals were reported as suppressors of mitotic division of plant cells. Growth parameters, i.e., root, shoot, and pod length and biomass, number of leaves and pods, increased at the lower rates of 10 and 20 g/kg; growth parameters decreased at the higher rates of 30 and 40 g/kg. The negative effects of a high rate of SS application on plant growth may refer to the toxic effects of heavy metals which include nutritional imbalance caused by the displacement of essential cations with heavy metals, interaction with sulfhydryl groups of functional proteins, decreasing chlorophyll content, affecting hormonal balance and generating reactive oxygen species in plant cells. SS salinity and organic content were greater than that of the cultivated soil used in the experiment. The accumulation trend of heavy metals in plant portions differed according to the amendment rate. Heavy metals in the postharvest soil samples increased for all SS application rates except Fe and Mo. The significant increase in Cd content started at the lowest amendment rate 10 g/kg; for Co, Mn, and Pb, the significant increase was detected at the highest amendment rate (40 g/kg). Generally, all heavy metals increased significantly in portions of *P. sativum* except Cd in the shoot. At an amendment rate of 10 g/kg, Co in the shoot and root, Cr in the fruit, Cu in the root, Fe in the fruit, Mn in the shoot and fruit, Mo in the fruit, Pb in the shoot, and Zn in the fruit were elevated significantly. In contrast, the concentrations of Cd in the fruit, Cr in the root, Cu in the shoot, Fe in the shoot and root, Ni in the fruit and root, Pb in the fruit and root, and Zn in the root significantly increased only at the highest rate of 40 g/kg. The regression *R*^2^ value was 0.927 for the micronutrient Mn in the pod and 0.154 for the heavy metal Cd in the shoot. Low metal translocation was detected for all measured heavy metals. Bioaccumulation and translocation factors were > 1 for Mo and the bioaccumulation of Pb was > 1. The suitable level of SS application into agricultural soil as fertilizer may differ according to the soil pH and the grown plant species. Considering the current study, the application rate of SS as a fertilizer for pea plants should not exceed 20 g/kg to avoid environmental and health risks for animals and humans. 

## Figures and Tables

**Table 1:** Selected chemical properties of sewage sludge and agricultural soil used in the pot experiment (means ± standard error, *n* = 3).

Properties	Sewage Sludge		Agricultural Soil	
	Measured Values	Permissible Limits *	Measured Values	Average Normal Limits **
Salinity (mS/cm)	1.39 ± 0.10	NA	0.07 ± 0.00	NA
pH	6.98 ± 0.02	NA	8.68 ± 0.02	NA
Organic matter (%)	65.0 ± 0.9	NA	0.9 ± 0.2	NA
Cd (mg/kg)	1.17 ± 0.08	20.0–40.0	2.91 ± 0.05	3.0
Co (mg/kg)	25.86 ± 1.31	-	35.49 ± 1.13	35.0
Cr (mg/kg)	176.18 ± 1.94	900.0	134.34 ± 0.66	125.0
Cu (mg/kg)	162.56 ± 2.32	1000.0–1750.0	15.01 ± 0.57	105.0
Fe (mg/g)	24.41 ± 0.52	-	42.37 ± 0.53	39.2
Mn (mg/kg)	560.70 ± 9.81	-	677.27 ± 3.23	775.0
Mo (mg/kg)	0.91 ± 0.04	-	1.06 ± 0.01	7.0
Ni (mg/kg)	138.73 ± 3.71	300.0–400.0	68.09 ± 3.70	40.0
Pb (mg/kg)	671.11 ± 6.22	750.0–1200.0	3.51 ± 0.39	160.0
Zn (mg/kg)	667.62 ± 13.44	2500.0–4000.0	77.18 ± 1.94	200.0

*: He et al. (2005), **: Kabata-Pendias (2011), NA: not applicable.

**Table 2:** Selected chemical properties of soil at different sewage sludge amendment rates after harvesting *Pisum sativum* that had been grown for 57 days (means ± standard error, *n* = 6).

Properties	Sewage Sludge Amendment Rate (g/kg)					*F*-value	Maximum Permissible Limits in Agricultural Soil ^†^
	0	10	20	30	40		
Salinity (mS/cm)	0.35 ± 0.05 ^a^	0.47 ± 0.03 ^ab^	0.52 ± 0.02 ^ab^	0.55 ± 0.05 ^b^	0.58 ± 0.07 ^b^	4.0 *	NA
pH	8.38 ± 0.07 ^e^	7.88 ± 0.03 ^d^	7.63 ± 0.03 ^c^	7.37 ± 0.03 ^b^	7.09 ± 0.01 ^a^	157.2 ***	NA
Organic matter (%)	1.20 ± 0.15 ^a^	3.04 ± 0.11 ^b^	4.66 ± 0.18 ^c^	6.85 ± 0.26 ^d^	6.78 ± 0.06 ^d^	215.9 ***	NA
Cd (mg/kg)	2.21 ± 0.17 ^a^	3.29 ± 0.23 ^b^	3.39 ± 0.09 ^b^	3.64 ± 0.03 ^b^	3.65 ± 0.09 ^b^	17.9 ***	1.0–5.0
Co (mg/kg)	26.21 ± 0.27 ^a^	26.56 ± 0.10 ^a^	28.60 ± 1.88 ^ab^	28.70 ± 0.22 ^ab^	30.86 ± 0.31 ^b^	4.7 **	20.0–50.0
Cr (mg/kg)	131.34 ± 3.72 ^a^	134.33 ± 4.23 ^ab^	139.82 ± 1.40 ^ab^	145.89 ± 1.13 ^b^	146.72 ± 4.41 ^b^	4.3 **	50.0–200.0
Cu (mg/kg)	16.51 ± 0.64 ^a^	19.97 ± 1.30 ^a^	29.37 ± 0.78 ^b^	31.34 ± 3.03 ^b^	31.70 ± 0.09 ^b^	20.9 ***	60.0–150.0
Fe (mg/g)	38.16 ± 0.39 ^a^	39.88 ± 0.34 ^a^	40.44 ± 0.82 ^a^	42.74 ± 1.68 ^a^	42.76 ± 6.05 ^a^	0.5 ^ns^	20.0–40.0 ^††^
Mn (mg/kg)	587.5 ± 2.7 ^a^	607.6 ± 22.3 ^ab^	616.9 ± 7.2 ^ab^	621.6 ± 6.3 ^ab^	641.2 ± 2.0 ^b^	3.2 *	<450.0 ^‡^
Mo (mg/kg)	1.05 ± 0.12 ^a^	1.07 ± 0.10 ^a^	1.10 ± 0.01 ^a^	1.18 ± 0.02 ^a^	1.19 ± 0.06 ^a^	1.1 ^ns^	4.0–10.0
Ni (mg/kg)	31.30 ± 0.24 ^a^	32.11 ± 1.25 ^a^	32.71 ± 0.12 ^a^	34.88 ± 0.04 ^b^	35.02 ± 0.36 ^b^	7.8 ***	20.0–60.0
Pb (mg/kg)	3.27 ± 0.15 ^a^	3.43 ± 0.04 ^a^	3.85 ± 0.11 ^a^	3.97 ± 0.25 ^a^	4.73 ± 0.24 ^b^	10.5 ***	20.0–30.0
Zn (mg/kg)	71.18 ± 0.72 ^a^	72.42 ± 3.70 ^a^	100.70 ± 8.67 ^b^	102.08 ± 0.03 ^b^	108.52 ± 2.02 ^b^	16.9 ***	100.0–300.0

*F*-values represent one-way ANOVA, degrees of freedom (*df*) = 4. Means in the same row followed by different letters are significantly different at *p* < 0.05 according to Tukey’s HSD test. *: *p* < 0.05, **: *p* < 0.01, ***: *p* < 0.001, *ns*: not significant (i.e., *p* > 0.05), NA: not applicable, ^†^ reviewed by Kabata-Pendias (2011), ^††^ Cornell and Schwertmann (2003), ^‡^ Adriano (2001).

**Table 3:** Effects of different amendment rates of sewage sludge on heavy metal concentrations (mg/ kg) in fruits, shoots, and roots of *Pisum sativum* plants that were harvested after 57 days (means ± standard error, *n* = 6).

Metal	Tissue	Sewage Sludge Amendment Rate (g/kg)					*F*-Value	Safe Limit +	Phytotoxic Range ‡
		0	10	20	30	40			
Cd	Fruit	0.21 ± 0.03 ^a^	0.24 ± 0.03 ^a^	0.24 ± 0.00 ^a^	0.25 ± 0.00 ^a^	0.51 ± 0.00 ^b^	44.4 ***	0.3	5–30
	Shoot	0.24 ± 0.00 ^a^	0.26 ± 0.02 ^a^	0.26 ± 0.02 ^a^	0.27 ± 0.02 ^a^	0.30 ± 0.02 ^a^	1.5 ^ns^		
	Root	0.42 ± 0.03 ^a^	0.53 ± 0.08 ^a^	0.68 ± 0.09 ^ab^	0.80 ± 0.01 ^b^	0.81 ± 0.09 ^b^	6.0 **		
Co	Fruit	1.60 ± 0.01 ^a^	1.79 ± 0.13 ^ab^	1.93 ± 0.03 ^b^	3.17 ± 0.06 ^c^	4.60 ± 0.01 ^d^	399.6 ***	-	30–40
	Shoot	0.84 ± 0.10 ^a^	1.44 ± 0.07 ^b^	1.51 ± 0.03 ^b^	1.74 ± 0.12 ^bc^	2.03 ± 0.09 ^c^	25.6 ***		
	Root	9.66 ± 0.24 ^a^	13.16 ± 1.69 ^b^	13.96 ± 0.59 ^b^	14.54 ± 0.12 ^b^	14.76 ± 0.30 ^b^	6.5 **		
Cr	Fruit	1.44 ± 0.02 ^a^	2.52 ± 0.21 ^b^	4.09 ± 0.25 ^c^	4.84 ± 0.16 ^d^	5.33 ± 0.07 ^d^	94.9 ***	5	10–100
	Shoot	2.43 ± 0.06 ^a^	2.85 ± 0.13 ^ab^	3.63 ± 0.03 ^abc^	4.09 ± 0.74 ^bc^	5.14 ± 0.49 ^c^	7.0 **		
	Root	30.64 ± 4.64 ^a^	43.15 ± 7.29 ^a^	44.29 ± 3.38 ^a^	45.41 ± 3.58 ^a^	63.39 ± 2.32 ^b^	6.6 **		
Cu	Fruit	4.97 ± 0.02 ^a^	6.08 ± 0.04 ^a^	6.94 ± 0.03 ^a^	9.35 ± 0.83 ^b^	11.83 ± 0.88 ^c^	26.1 ***	40	20–100
	Shoot	3.48 ± 0.48 ^a^	4.49 ± 0.27 ^a^	4.61 ± 0.04 ^a^	4.70 ± 0.01 ^a^	8.49 ± 0.52 ^b^	32.7 ***		
	Root	4.48 ± 0.22 ^a^	14.66 ± 0.22 ^b^	16.00 ± 1.96 ^bc^	17.03 ± 1.03 ^bc^	18.77 ± 0.19 ^c^	31.4 ***		
Fe	Fruit	112.3 ± 4.2 ^a^	227.7 ± 39.9 ^b^	264.1 ± 45.3 ^bc^	281.9 ± 5.0 ^bc^	347.7 ± 11.9 ^c^	9.8 ***	450	>1000
	Shoot	266.9 ± 28.7 ^a^	278.5 ± 43.9 ^a^	364.7 ± 33.2 ^a^	370.4 ± 76.9 ^a^	647.7 ± 53.7 ^b^	9.4 ***		
	Root	8405.6 ± 1152.4 ^a^	12,189.6 ± 2123.5 ^a^	12,212.5 ± 890.9 ^a^	12,290.8 ± 910.3 ^a^	17,479.6 ± 251.6 ^b^	6.9 **		
Mn	Fruit	43.2 ± 0.9 ^a^	85.3 ± 6.9 ^b^	93.5 ± 0.9 ^b^	167.0 ± 5.0 ^c^	218.2 ± 3.8 ^d^	274.8 ***	-	>400
	Shoot	95.2 ± 10.5 ^a^	152.9 ± 3.5 ^b^	158.7 ± 2.5 ^b^	164.4 ± 0.6 ^b^	271.1 ± 1.2 ^c^	157.0 ***		
	Root	366.7 ± 25.7 ^a^	380.1 ± 2.1 ^a^	391.3 ± 15.0 ^a^	416.1 ± 0.5 ^a^	454.1 ± 61.9 ^a^	1.3 ^ns^		
Mo	Fruit	3.23 ± 0.12 ^a^	4.02 ± 0.01 ^b^	4.21 ± 0.07 ^b^	9.83 ± 0.13 ^c^	21.16 ± 0.21 ^d^	3632.6 ***	10	135
	Shoot	2.31 ± 0.11 ^a^	2.34 ± 0.12 ^a^	4.88 ± 0.59 ^b^	5.20 ± 0.65 ^b^	15.43 ± 0.84 ^c^	97.9 ***		
	Root	1.87 ± 0.38 ^a^	3.16 ± 0.45 ^ab^	3.99 ± 0.89 ^ab^	4.88 ± 0.45 ^bc^	6.85 ± 1.14 ^c^	6.7 **		
Ni	Fruit	5.17 ± 0.04 ^a^	5.20 ± 0.09 ^a^	5.88 ± 0.60 ^ab^	6.20 ± 0.07 ^ab^	6.57 ± 0.07 ^b^	4.9 **	20	40–246
	Shoot	2.07 ± 0.31 ^a^	2.11 ± 0.08 ^a^	2.21 ± 0.01 ^a^	3.09 ± 0.13 ^b^	3.68 ± 0.36 ^b^	10.3 ***		
	Root	21.07 ± 1.42 ^a^	22.72 ± 1.01 ^a^	23.36 ± 3.11 ^ab^	24.49 ± 0.59 ^ab^	29.30 ± 0.08 ^b^	3.7 *		
Pb	Fruit	0.39 ± 0.02 ^a^	0.41 ± 0.02 ^a^	0.44 ± 0.03 ^a^	0.49 ± 0.05 ^a^	1.02 ± 0.01 ^b^	77.4 ***	5	30–300
	Shoot	0.38 ± 0.05 ^a^	3.44 ± 0.21 ^b^	4.09 ± 0.31 ^b^	5.90 ± 0.44 ^c^	6.03 ± 0.32 ^c^	59.6 ***		
	Root	5.05 ± 0.95 ^a^	6.36 ± 0.47 ^a^	6.55 ± 0.56 ^ab^	7.83 ± 1.02 ^ab^	9.31 ± 0.43 ^b^	4.9 **		
Zn	Fruit	29.8 ± 0.3 ^a^	32.0 ± 0.5 ^b^	33.2 ± 0.3 ^c^	37.2 ± 0.2 ^d^	63.7 ± 0.2 ^e^	1925.9 ***	60	100–500
	Shoot	13.6 ± 1.4 ^a^	18.6 ± 1.0 ^ab^	19.3 ± 1.0 ^ab^	24.8 ± 0.1 ^b^	39.2 ± 3.5 ^c^	29.6 ***		
	Root	51.3 ± 6.1 ^a^	62.9 ± 1.0 ^a^	69.7 ± 5.4 ^a^	69.8 ± 1.8 ^a^	148.2 ± 20.3 ^b^	15.5 ***		

*F*-values represent one-way ANOVA, degrees of freedom (*df*) = 4. Means in the same row followed by different letters are significantly different at *p* < 0.05, according to Tukey’s HSD test. *: *p* < 0.05, **: *p* < 0.01, ***: *p* < 0.001, *ns*: not significant (i.e., *p* > 0.05), +: FAO/WHO standard [45] (Codex Alimentarious Commission 2011), ‡: [34] Kabata-Pendias (2011).

**Table 4:** Linear regression equations of the form *y* = *a* + *bx*, where *y* represents the heavy metal concentration (mg/kg) in *Pisum sativum* tissue harvested after 57 days, and *x* is the sewage sludge amendment rate (g/kg).

*Y*		*A*	*SE*	*B*	*SE*	*R* ^2^	*p*
Cd	Fruit	0.168	0.027	0.006	0.001	0.530	0.000
	Shoot	0.241	0.014	0.001	0.001	0.154	0.032
	Root	0.437	0.052	0.010	0.002	0.461	0.000
Co	Fruit	1.142	0.152	0.074	0.006	0.833	0.000
	Shoot	0.978	0.073	0.027	0.003	0.743	0.000
	Root	10.899	0.666	0.116	0.027	0.393	0.000
Cr	Fruit	1.624	0.153	0.101	0.006	0.904	0.000
	Shoot	2.299	0.297	0.066	0.012	0.518	0.000
	Root	31.819	3.617	0.678	0.148	0.429	0.000
Cu	Fruit	4.434	0.433	0.170	0.018	0.767	0.000
	Shoot	3.103	0.395	0.102	0.016	0.591	0.000
	Root	7.998	1.098	0.310	0.045	0.630	0.000
Fe	Fruit	141.751	21.614	5.250	0.882	0.558	0.000
	Shoot	214.941	42.742	8.536	1.745	0.461	0.000
	Root	8865.751	993.829	182.494	40.573	0.419	0.000
Mn	Fruit	35.101	5.589	4.316	0.228	0.927	0.000
	Shoot	95.846	8.908	3.632	0.364	0.781	0.000
	Root	359.479	22.653	2.109	0.925	0.157	0.030
Mo	Fruit	0.155	1.086	0.417	0.044	0.759	0.000
	Shoot	0.212	0.934	0.291	0.038	0.676	0.000
	Root	1.815	0.539	0.117	0.022	0.502	0.000
Ni	Fruit	5.046	0.207	0.038	0.008	0.419	0.000
	Shoot	1.791	0.183	0.042	0.007	0.530	0.000
	Root	20.540	1.234	0.182	0.050	0.319	0.001
Pb	Fruit	0.278	0.052	0.014	0.002	0.595	0.000
	Shoot	1.218	0.308	0.138	0.013	0.810	0.000
	Root	5.020	0.540	0.100	0.022	0.421	0.000
Zn	Fruit	24.582	2.313	0.731	0.094	0.681	0.000
	Shoot	11.580	1.722	0.575	0.070	0.705	0.000
	Root	40.256	9.673	2.006	0.395	0.480	0.000

*SE*: standard error, *n* = 30.

**Table 5:** Bioaccumulation factors (BFs), from soil to roots, and translocation factors (TFs), from roots to fruits and shoots, of heavy metals in *Pisum sativum* grown in soil with different sewage sludge amendment rates (means ± standard error, *n* = 6).

Metal	Factor	Sewage Sludge Amendment rate (g/kg)					*F*-Value
		0	10	20	30	40	
Cd	BF	0.194 ± 0.008 ^ab^	0.156 ± 0.012 ^a^	0.196 ± 0.024 ^ac^	0.219 ± 0.005 ^bc^	0.219 ± 0.019 ^bc^	2.9 *
	TF*_shoot_*	0.582 ± 0.036 ^b^	0.566 ± 0.108 ^b^	0.426 ± 0.053 ^ab^	0.333 ± 0.028 ^a^	0.384 ± 0.013 ^ab^	3.7 *
	TF*_fruit_*	0.501 ± 0.071 ^ab^	0.497 ± 0.083 ^ab^	0.404 ± 0.059 ^a^	0.308 ± 0.003 ^a^	0.675 ± 0.077 ^b^	4.3 **
Co	BF	0.368 ± 0.005 ^a^	0.495 ± 0.063 ^a^	0.506 ± 0.054 ^a^	0.507 ± 0.001 ^a^	0.478 ± 0.007 ^a^	2.5 ^ns^
	TF*_shoot_*	0.086 ± 0.009 ^a^	0.116 ± 0.010 ^ab^	0.108 ± 0.003 ^ab^	0.120 ± 0.009 ^b^	0.138 ± 0.009 ^b^	5.3 **
	TF*_fruit_*	0.166 ± 0.003 ^b^	0.141 ± 0.009 ^a^	0.139 ± 0.008 ^a^	0.218 ± 0.006 ^c^	0.312 ± 0.007 ^d^	113.6 ***
Cr	BF	0.239 ± 0.042 ^a^	0.331 ± 0.065 ^ab^	0.316 ± 0.021 ^ab^	0.312 ± 0.027 ^ab^	0.432 ± 0.009 ^b^	3.3 *
	TF*_shoot_*	0.091 ± 0.016 ^a^	0.074 ± 0.009 ^a^	0.084 ± 0.007 ^a^	0.086 ± 0.009 ^a^	0.080 ± 0.005 ^a^	0.4 ^ns^
	TF*_fruit_*	0.053 ± 0.008 ^a^	0.063 ± 0.006 ^ab^	0.093 ± 0.001 ^c^	0.112 ± 0.012 ^c^	0.085 ± 0.004 ^bc^	10.3 ***
Cu	BF	0.276 ± 0.024 ^a^	0.746 ± 0.038 ^b^	0.556 ± 0.082 ^b^	0.587 ± 0.089 ^b^	0.592 ± 0.006 ^b^	8.8 ***
	TF*_shoot_*	0.759 ± 0.071 ^c^	0.308 ± 0.023 ^ab^	0.309 ± 0.035 ^ab^	0.281 ± 0.017 ^a^	0.451 ± 0.023 ^b^	26.4 ***
	TF*_fruit_*	1.122 ± 0.055 ^c^	0.415 ± 0.008 ^a^	0.468 ± 0.056 ^a^	0.545 ± 0.016 ^ab^	0.628 ± 0.040 ^b^	49.9 ***
Fe	BF	0.222 ± 0.032 ^a^	0.304 ± 0.051 ^ab^	0.305 ± 0.028 ^ab^	0.294 ± 0.033 ^ab^	0.452 ± 0.069 ^b^	3.5 *
	TF*_shoot_*	0.038 ± 0.009 ^a^	0.023 ± 0.001 ^a^	0.030 ± 0.001 ^a^	0.029 ± 0.004 ^a^	0.037 ± 0.003 ^a^	1.9 ^ns^
	TF*_fruit_*	0.014 ± 0.002 ^a^	0.019 ± 0.000 ^ab^	0.021 ± 0.002 ^b^	0.024 ± 0.002 ^b^	0.020 ± 0.001 ^ab^	4.7 **
Mn	BF	0.625 ± 0.046 ^a^	0.629 ± 0.019 ^a^	0.639 ± 0.032 ^a^	0.669 ± 0.007 ^a^	0.709 ± 0.099 ^a^	0.5 ^ns^
	TF*_shoot_*	0.256 ± 0.011 ^a^	0.403 ± 0.011 ^a^	0.407 ± 0.009 ^a^	0.395 ± 0.002 ^a^	0.656 ± 0.087 ^b^	13.3 ***
	TF*_fruit_*	0.122 ± 0.011 ^a^	0.224 ± 0.017 ^a^	0.241 ± 0.012 ^a^	0.401 ± 0.012 ^b^	0.536 ± 0.081 ^b^	18.2 ***
Mo	BF	1.697 ± 0.174 ^a^	2.942 ± 0.420 ^ab^	3.591 ± 0.776 ^abc^	4.121 ± 0.346 ^bc^	5.609 ± 0.687 ^c^	7.5 ***
	TF*_shoot_*	1.642 ± 0.399 ^ab^	0.854 ± 0.159 ^a^	1.845 ± 0.559 ^ab^	1.176 ± 0.241 ^ab^	2.496 ± 0.292 ^b^	3.1 *
	TF*_fruit_*	2.256 ± 0.531 ^ab^	1.420 ± 0.205 ^a^	1.425 ± 0.332 ^a^	2.089 ± 0.166 ^ab^	3.554 ± 0.560 ^b^	4.9 **
Ni	BF	0.675 ± 0.051 ^a^	0.719 ± 0.059 ^a^	0.712 ± 0.093 ^a^	0.702 ± 0.017 ^a^	0.837 ± 0.010 ^a^	1.3 ^ns^
	TF*_shoot_*	0.095 ± 0.008 ^a^	0.093 ± 0.001 ^a^	0.104 ± 0.014 ^ab^	0.126 ± 0.003 ^b^	0.125 ± 0.012 ^ab^	3.2 *
	TF*_fruit_*	0.251 ± 0.015 ^a^	0.230 ± 0.006 ^a^	0.257 ± 0.009 ^a^	0.254 ± 0.009 ^a^	0.224 ± 0.003 ^a^	2.6 ^ns^
Pb	BF	1.624 ± 0.360 ^a^	1.862 ± 0.154 ^a^	1.730 ± 0.195 ^a^	2.092 ± 0.382 ^a^	2.014 ± 0.183 ^a^	0.5 ^ns^
	TF*_shoot_*	0.081 ± 0.007 ^a^	0.557 ± 0.059 ^b^	0.668 ± 0.109 ^b^	0.877 ± 0.209 ^b^	0.658 ± 0.054 ^b^	7.1 **
	TF*_fruit_*	0.095 ± 0.019 ^a^	0.067 ± 0.008 ^a^	0.071 ± 0.010 ^a^	0.075 ± 0.018 ^a^	0.111 ± 0.005 ^a^	1.9 ^ns^
Zn	BF	0.726 ± 0.094 ^a^	0.884 ± 0.059 ^a^	0.743 ± 0.118 ^a^	0.683 ± 0.017 ^a^	1.386 ± 0.213 ^b^	5.9 **
	TF*_shoot_*	0.267 ± 0.006 ^a^	0.296 ± 0.020 ^ab^	0.292 ± 0.037 ^ab^	0.357 ± 0.008 ^b^	0.274 ± 0.014 ^a^	3.1 *
	TF*_fruit_*	0.629 ± 0.081 ^a^	0.508 ± 0.001 ^a^	0.493 ± 0.043 ^a^	0.535 ± 0.012 ^a^	0.474 ± 0.064 ^a^	1.5 ^ns^
*F-value_BF_*		17.7 ***	33.4 ***	15.6 ***	53.2 ***	46.0 ***	
*F-value_TFshoot_*		15.1 ***	17.3 ***	8.7 ***	13.4 ***	54.3 ***	
*F-value_TFfruit_*		16.4 ***	34.5 ***	14.0 ***	126.4 ***	32.7 ***	

*F*-values represent one-way ANOVA, degrees of freedom (*df*) = 4. Means in the same row followed by different letters are significantly different at *p* < 0.05, according to Tukey’s HSD test. *: *p* < 0.05, **: *p* < 0.01, ***: *p* < 0.001, *ns*: not significant (i.e., *p* > 0.05).
